# The benefits of being toxic to deter predators depends on prey body size

**DOI:** 10.1093/beheco/arw086

**Published:** 2016-06-21

**Authors:** Karen E. Smith, Christina G. Halpin, Candy Rowe

**Affiliations:** Centre for Behaviour and Evolution, Institute of Neuroscience, Newcastle University, Henry Wellcome Building, Framlington Place, Newcastle upon Tyne NE2 4HH, UK

**Keywords:** Batesian mimicry, cognition, nutrient-toxin trade-off, predator, toxicity, warning signal.

## Abstract

Many prey have evolved toxins as a defense against predation. Those species that advertise their toxicity to would-be predators with conspicuous warning signals are known as “aposematic.” Investment in toxicity by aposematically signaling prey is thought to underpin how aversive prey are to predators; increasing toxicity means that predators learn to avoid prey faster and attack them at lower rates. However, predators’ foraging decisions on aposematic prey are determined not only by their toxicity, but also by their nutrient content: predators can trade-off the costs of ingesting toxin with the benefits of acquiring nutrients. Prey body size is a cue that positively correlates with nutrient content, and that varies within and between aposematic species. We predicted that a dose of quinine (known to be toxic to birds) would be a more effective deterrent to avian predators when prey were small compared with when they were large, and that the benefits of possessing toxin would be greater for small-bodied prey. Using an established laboratory protocol of European starlings (*Sturnus vulgaris*) foraging on mealworms (*Tenebrio molitor*), we found evidence for increased protection from a dose of quinine for small-bodied compared with large-bodied prey. This shows that larger prey need more toxin to attain the same level of defense as smaller prey, which has implications for the evolution of aposematism and mimicry.

## Lay Summary

If you’re a toxic insect trying to avoid being eaten by a foraging bird, it might be better to be small. The ways in which birds make decisions about what to eat are based on the benefits of eating nutrients and the unpleasant consequences of ingesting toxins. Larger prey will need to contain more toxins in order to deter predators to the same extent as small prey. Being big isn’t always better.

## INTRODUCTION

Many aposematic prey species arm themselves with toxins that are harmful or unpleasant to predators, and advertise those defenses using a variety of conspicuous warning signals (Poulton 1890; Mappes et al. 2005; Halpin et al. 2013). Although warning signals can be costly in terms of increased detection, they are also particularly salient to predators, allowing them to quickly learn about and identify toxic prey and reduce their attack rates on them (Gittleman and Harvey 1980; Alatalo and Mappes 1996; Lindström et al. 1999). Therefore, aposematic signals appear to have been selected to take advantage of the cognitive processes of predators, and in particular, how they learn about prey and make dietary decisions in a complex world (Mappes et al. 2005; Stevens and Ruxton 2012).

Traditionally, signal design and toxin content have been considered to be the 2 intrinsic properties of aposematic prey that determine the efficacy of the defense and enhance prey survival. Empirical studies following this approach have been important in demonstrating how warning signals are designed to enhance learned and unlearned avoidance in predators (e.g., Gittleman and Harvey 1980; Roper and Redston 1987; Rowe and Guilford 1996; Lindström et al. 1999; Aronsson and Gambarale-Stille 2009; Svádová et al. 2009), and how increasing the detectability, amount, or variability of toxicity can increase predator aversions (e.g., Skelhorn and Rowe 2006a, 2006b, 2006c; Barnett et al. 2012, 2014). However, a recent study has shown that there is another intrinsic factor that affects foraging predators’ decisions to attack aposematic prey: the nutritional content of prey. It is sometimes easy to forget that aposematic prey contain valuable nutrients as well as toxins, and what predators know about the nutrient content of toxic prey can also affect their decisions to attack and eat them (Halpin et al. 2014). In the wild, predators may need to attack and ingest aposematic prey in order to acquire the nutrients that they contain, particularly when energetically stressed (Marshall 1908; Sexton et al. 1966; Fink et al. 1983; Brower and Calvert 1985; Chai 1986; Barnett et al. 2007, 2012; Chatelain et al. 2013). Currently, we know very little about how predators learn about and integrate information about nutrient and toxin content in their foraging decisions on aposematic prey.

One cue that a predator could use to assess nutritional value of aposematic prey is body size: body size correlates with nutrient content, and is relatively easy for predators to evaluate and use in their foraging decisions (e.g., Wiegert 1965; Barnard and Brown 1981; Lease and Wolf 2011). There is increasing interest in the role that body size might play in the effectiveness of aposematism as a defense strategy. For example, larger bodies can carry larger signals, which could make aposematic prey more detectable (Mänd et al. 2007; Remmel and Tammaru 2009), but also enhance predator aversions (Lindstedt et al. 2008). However, larger bodies may also make prey more profitable to predators, particularly when handling times associated with larger prey are not increased (Charnov 1976). Under these conditions, larger prey may need to invest more in toxicity to achieve the same level of defense as smaller prey (Speed and Ruxton 2014). Comparative studies provide some evidence that variation in body size both within and between aposematic species can positively correlate with toxin concentration (Hagman and Forsman 2003; Kraemer et al. 2015). These results suggest that larger aposematic prey need more toxin to achieve the same level of protection from predators as smaller prey, and that they will have a higher predation risk compared with smaller prey when they contain the same amount of toxin. Although seemingly intuitive, there has been no direct test of this prediction.

Using an established protocol of European starlings (*Sturnus vulgaris*) foraging on mealworms (*Tenebrio molitor*), we tested if small and large insect prey achieved the same level of protection against an avian predator when they contained the same amount of toxin. Birds were trained to discriminate between an undefended prey population (including large and small individuals), and 2 populations of defended prey that carried distinct visual signals. All defended prey were injected with the same amount of quinine (a mild aversant to birds [Alcock 1970; Alatalo and Mappes 1996; Skelhorn and Rowe 2006a]), but one population was small-bodied and the other large-bodied. This design allowed us to measure not just differences in attack rates on defended prey with different sized bodies (e.g., Smith et al. 2014), but the advantage of being defended relative to being undefended for a given body size. We predicted that whilst birds would learn to reduce their attacks on both types of defended prey, the benefits of being toxic would be greater for small prey compared with large prey.

## METHODS

### Subjects and housing

Ten (5 male, 5 female) European Starlings (*S. vulgaris*) were caught under license from Natural England (20103688), and housed in pairs in adjoining individual wire cages (45×75×45cm). A solid wood divider was used to separate the birds for the experimental sessions. Birds were maintained on a 10L:14D cycle at 16–17 °C. Water was provided ad lib. Zoofood pheasant breeder pellets were freely available except during experimental sessions. At the end of the experiment, birds were weighed and returned to a free flight aviary before being health checked by a vet and released at their site of capture. The experiment was conducted under Local Ethical Committee approval (ERC Project ID: 266), and in accordance with ASAB’s Guidelines for the Treatment of Animals in Behavioural Research and Teaching.

### Training sessions

Birds were initially trained to eat 2 sizes of mealworms (*T. molitor*): small (0.15–0.17g) and large (0.31–0.33g). In mealworms, body size is known to correlate with nutrient content (Finke 2002). On 3 consecutive days, each bird received a single training session. Birds were separated in their home cage using an opaque divider, and food was removed 75min before the start of a session. Five minutes prior to the start of a training session, the front of the cage was covered with a white curtain to visually isolate subjects. Cameras placed at the side of the cages were used to observe the birds. A random sequence of 12 small and 12 large mealworm prey were presented to each bird in a clear petri dish (38mm diameter) that was inserted through a small door at the base of the cage onto the white floor. Birds were allowed 1min to attack each mealworm before it was removed. If the bird attacked the mealworm, the petri dish was removed immediately after the attack. There were 3min between each mealworm presentation. In order to be included in the experimental analysis, birds were required to reach the criterion of eating at least 80% of the prey presented in 2 consecutive training sessions. Only 7 birds (3 males, 4 females) achieved that criterion.

### Learning sessions

Ten learning sessions followed the same methodology as the training sessions, except that birds received 3 prey types with visually distinct signals in each session: small defended, large defended and undefended prey. Undefended prey included small and large mealworms, which were potentially visually discriminable prior to attack. Each bird received 24 mealworms in a session; 8 of each signaling prey type. Small defended prey were small mealworms (0.15–0.17g) injected with 0.02mL 4% quinine solution. Large defended prey were large mealworms (0.31–0.33g) injected with 0.02mL 4% quinine solution. Undefended prey were small (0.15–0.17g) and large (0.31–0.33g) mealworms, 4 of each size, injected with 0.02mL water. We selected body sizes on the basis that larger mealworms are more nutritious (Finke 2002), and that starlings readily consume both mealworm sizes when palatable (i.e., the benefits of acquiring additional nutrients are not apparently offset by increased handling costs; Smith et al. 2014). The 3 prey types each had a visual signal, which was a disc of grey paper placed in the bottom of the petri dish under each mealworm. The shade of grey used differed depending on prey type. Shades of grey were selected on the basis that they were visually discriminable using the “grey scale slider bar” in Microsoft PowerPoint. For all birds, undefended prey had a 40% grey disc placed underneath. The 2 defended prey types were signaled by 65% and 15% grey discs, where 4 birds had 65% grey associated with the large undefended prey and 15% grey with the small defended prey, and the association was reversed for the other 3 birds. Therefore, all 3 prey types were visually distinguishable. In each prey presentation, birds were given 1min to eat the mealworm before it was removed, and there was 3min between presentations. The number of mealworms attacked and eaten was recorded; these measures were almost identical. Across all the experimental sessions, only 19 out of the 1137 mealworms that were attacked were not eaten by the birds. Therefore, we conducted our analyses using only one of these measures, the number of prey attacked, as our dependent variable.

### Simultaneous choice session

Following the final learning session, birds received an additional session that allowed us to investigate if they had learned to distinguish between large defended and large undefended prey on the basis of the visual signals. In previous experiments where starlings have learned to associate visual signals with the presence of toxicity or unpalatability, they have shown a strong avoidance of toxic and/or distasteful prey in simultaneous choice sessions (Barnett et al. 2007, 2012; Skelhorn and Rowe 2009; Halpin et al. 2014). Birds received a single simultaneous choice session, where they were given 16 presentations of a pair of petri dishes placed next to each other on the floor of the cage. In one petri dish was a large defended mealworm and in the other was a large undefended mealworm; both prey had their visual signal placed underneath. The session followed the same presentation protocol, although birds were allowed to attack just one of the mealworms before both dishes were removed from the cage. Birds attacked between 8 and 16 mealworms during the session, and the prey type that was attacked in each pair of presentations was recorded.

### Data analysis

When predators learn about aposematic prey, and associate the visual signal with toxicity, there are 2 distinct phases to the learning process. The first is where the attack rates on the aposematic prey change across sessions, as predators acquire information about the prey and associate their profitability with their visual appearance. This is known as the acquisition phase. The second phase is where the attack rates remain stable, and is where the predators use the information that they have acquired to make adaptive foraging decisions on the prey types available to them. This is known as the asymptotic phase (Pearce 2008; see also Skelhorn et al. 2016). Our first analysis aimed to establish the acquisition and asymptotic phases in order to analyze them separately. This was important as the behavior of birds in one phase can potentially mask differences in their behavior in another, particularly if one phase lasts much longer than another.

In order to measure the benefits of possessing quinine for each prey size in the acquisition and asymptotic phases, we needed to compare the number of attacks on small and large defended prey to the relevant size of undefended prey. Therefore, for each bird, we calculated the overall mortality for small and large undefended and defended prey, pooled across sessions separately for the acquisition and the asymptotic phases. Mortality was calculated as the number of each prey type attacked from those that were presented (see also e.g., Rowland et al. (2007) for use of the same measure).

## RESULTS

### Establishing the acquisition and asymptotic phases for subsequent analysis

**Figure 1:**
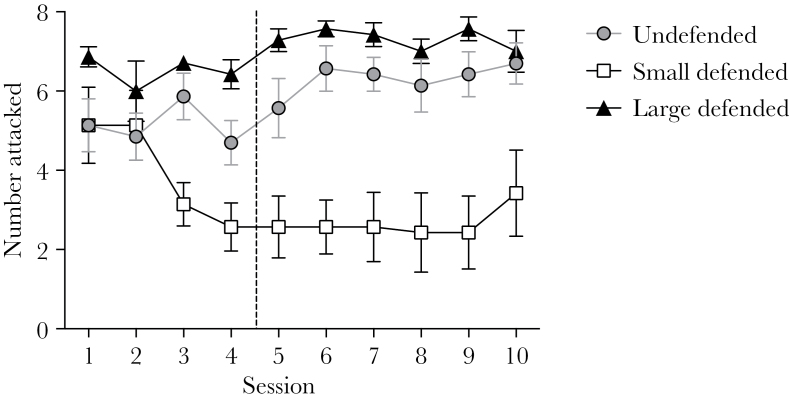
The mean (±SE) numbers of undefended, small defended and large defended prey attacked in each experimental session. Based on our analysis, we defined the acquisition phase as Sessions 1–4, and the asymptotic phase as Sessions 5–10. The dotted line delineates these 2 phases.

We initially analyzed the number of attacks on each prey type using a Generalized Estimating Equation (GEE) model (binomial distribution) in SPSS (v22) with prey type and session as main effects and bird as a random factor. There was a significant effect of session (χ^2^(6) = 1560, *P* < 0.001), prey type (χ^2^(2) = 48.2, *P* < 0.001) and an interaction between these 2 factors (χ^2^(6) = 81.3, *P* < 0.001; Figure 1). Because changes in the number attacks across the 10 sessions differed for each prey type (Figure 1), and it was important to establish the asymptote individually for every prey type, we investigated the effect of session on each prey type using separate GEEs with bird as a random factor, and repeated contrasts on the estimated means to investigate when there was no longer a significant change in the number attacked between subsequent sessions. There was a significant effect of session on the number of attacks on all prey types (all χ^2^(6) > 112.4, all *P* < 0.001), with the last significant difference between subsequent sessions occurring for the small defended prey between sessions 3 and 4 (χ^2^(1) = 4.31, *P* = 0.038). After session 4, there were no significant differences in the numbers of prey attacked for any prey type (all χ^2^(6) > 3.36, all *P* > 0.05). Therefore, we defined the acquisition phase as sessions 1–4 and the asymptotic phase as sessions 5–10.

### Acquisition phase (sessions 1–4)

**Figure 2:**
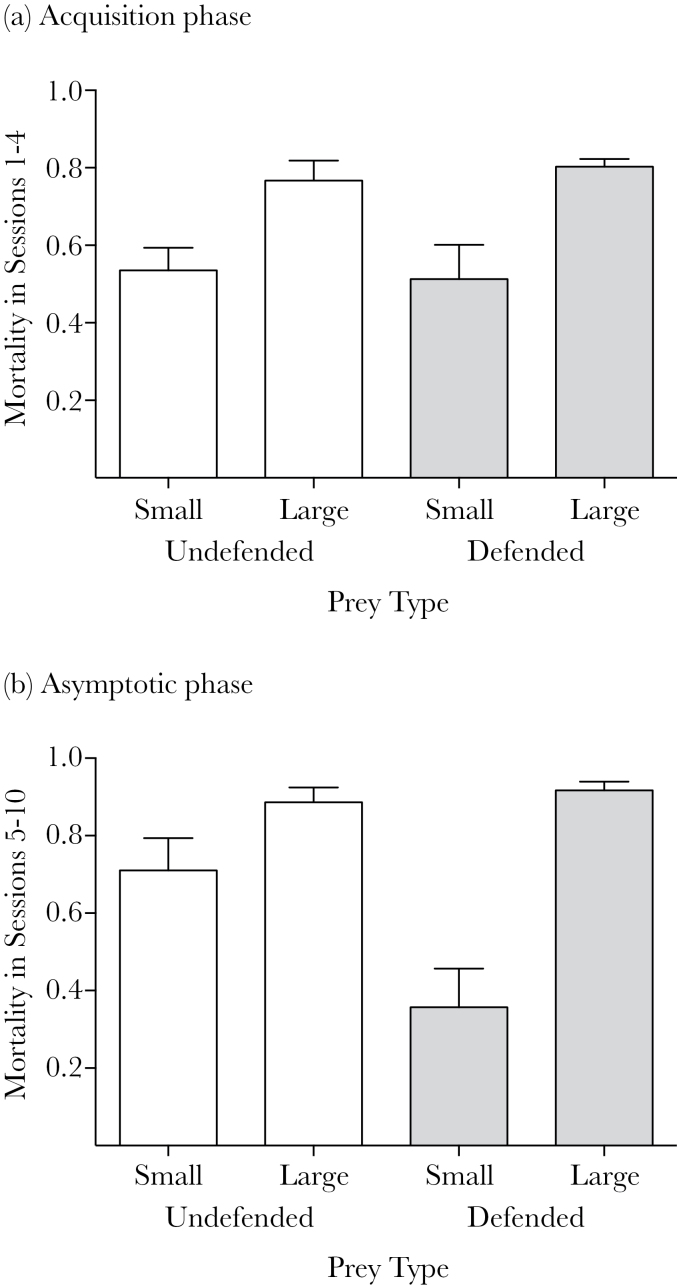
The mean (+SE) mortality of small and large undefended and defended prey in (a) the acquisition phase (Sessions 1–4), and (b) the asymptotic phase (Sessions 5–10).

We used a binomial GEE to explore the effects of defense and size on the mortality of each prey type, with bird as a random factor, and defense (undefended or defended) and size (small or large) as fixed factors. In this phase, birds attacked more large than small prey (χ^2^(1) = 28.8, *P* < 0.001), but there was no effect of defense (χ^2^(1) = 0.07, *P* > 0.05) and no interaction (χ^2^(1) = 0.53, *P* > 0.05; see Figure 2a).

### Asymptotic phase (sessions 5–10)

In this phase, our binomial GEE revealed a significant interaction between defense and size (χ^2^(1) = 21.86, *P* < 0.001), as well as significant main effects of size (χ^2^(1) = 44.19, *P* < 0.001) and defense (χ^2^(1) = 10.45, *P* < 0.005; see Figure 2b). Therefore, in support of our prediction, the quinine dose gave significantly more protection to the small compared with large prey in the asymptotic phase.

### Simultaneous choice session

**Figure 3:**
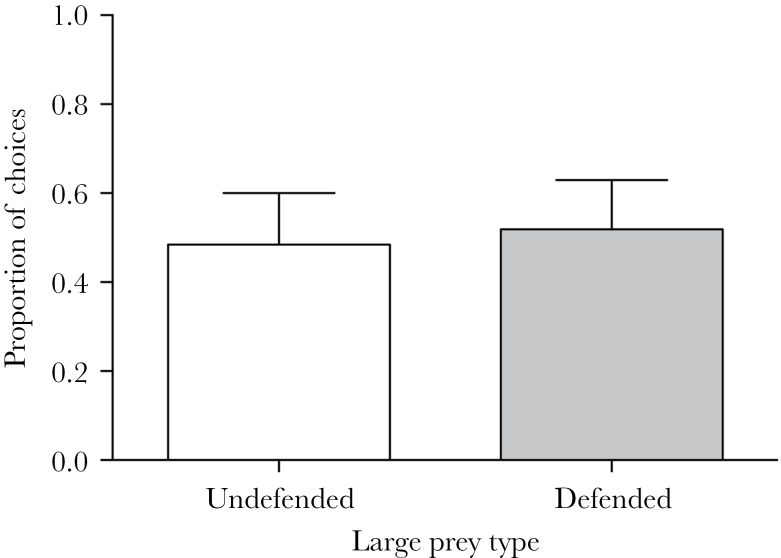
The mean (+SE) proportion of choices made for large undefended and large defended prey during the simultaneous choice session.

Given that birds attacked similar numbers of large undefended and defended prey in each session, we conducted a simultaneous choice session to test if birds would discriminate between defended and undefended large prey when given a simultaneous choice. For those presentations where they expressed a choice and attacked one of the mealworms, we found no difference in the proportion of large defended and undefended prey that they attacked (paired *t*-test: *t*_6_ = 0.42, *P* = 0.69; Figure 3). Therefore, birds were unable to distinguish, or chose not to distinguish, between defended and undefended large prey.

## DISCUSSION

Our data provide support for our main prediction that small-bodied prey benefit more than large-bodied prey when carrying the same amount of toxin. Birds only reduced their attacks on defended prey when they were small, with the numbers of attacks on large defended prey remaining high across all 10 sessions. These results are entirely consistent with the idea that predators are trading-off the benefits of acquiring nutrients with the costs of ingesting toxins (Speed 1993; Sherratt et al. 2004; Skelhorn et al. 2007; Halpin et al. 2014). It appears that large-bodied prey would indeed need to invest more in defense than small-bodied prey in order to acquire the same level of protection against predators (Speed and Ruxton 2014).

One surprising aspect of our results was that the large defended prey did not appear to receive any protection from their quinine defense. This is in contrast to a previous experiment where birds reduced their attacks on similarly-sized large defended prey, albeit more slowly than small defended prey (Smith et al. 2014). This could be due to the differences in the presentation methods between the 2 studies. In the previous experiment, small (0.15–0.17g) and large (0.31–0.33g) quinine-injected mealworms were also presented at equal frequency with medium-sized (0.22–0.25g) defended quinine-injected mealworms as a single prey type that shared the same visual signal (Smith et al. 2014). This could have meant that the large prey were more easily confused with smaller defended prey sizes, and that it was better for the birds to avoid all defended prey rather than risk ingesting a less rewarding medium or even small defended prey instead of a large one. If this was the case, it would lead to the intriguing possibility that large defended prey would benefit more from their defenses when they are less distinguishable from smaller and less energetically rewarding defended prey because of the uncertainty of the associated nutritional reward. This would be akin to the idea that birds find a toxic species more aversive if individuals vary in their toxicity, because the predator is less able to predict the toxin content of the prey and regulate its toxin intake (Barnett et al. 2014; Speed and Ruxton 2014). Our results suggest that this idea could be extended to consider how the variability in nutrient content could also be a crucial factor in risky decision-making on toxic prey.

However, there is a simpler explanation, which is that the large defended prey were not sufficiently aversive for the birds to learn to associate their visual signal with their toxin content. This idea is supported by the data from the simultaneous choice session, where birds were indiscriminate in their choices for large undefended and large defended prey when they were presented together. Although we cannot be sure that this was due to them not learning the difference between the 2 visual signals, in all previous studies using the same simultaneous choice protocol, we have found that starlings show strong avoidance for visual signals of prey that they have learned have higher toxin content or distastefulness (Barnett et al. 2007, 2012; Skelhorn and Rowe 2009; Halpin et al. 2014). This lack of discrimination was not due to the grey signals being indistinguishable or the toxin level being insufficient to elicit an aversion, because both of these were effective at reducing the number of attacks by the birds on small defended prey. What is possible is that the increased prey body size reduced the efficacy of the toxin once ingested, effectively making it less detectable and reducing the speed with which predators learned to associate the toxicity with the signal (Levine 1970). However, since we know that birds can use visual signals to avoid prey of this size that contain toxin (Smith et al. 2014), this seems an unlikely explanation.

This leads us then to consider how the ways in which birds might learn about the size or nutrient content of prey, affects what they learn about prey toxin content. One possibility is that the positive reinforcement from the nutrients in the large defended prey “overshadowed” the negative reinforcement from the quinine. Overshadowing is a term from the psychology literature used to describe when a more salient predictive cue gains more associative strength with an outcome than another (e.g., Pearce 1994). For example, if a dim light and a loud tone are used simultaneously to predict a food reward, the more salient acoustic cue gains more associative strength and affects the animal’s behavior more than the less salient visual cue. To our knowledge, the idea of overshadowing has not been extended to consider how the salience of the outcomes themselves might affect what is associated with a predictive cue. In the context of our experiment, it could be that the nutritional reward is simply more salient than the toxicity, and is associated more strongly with the visual signal than the prey’s quinine content. This could be a cognitive mechanism by which a single value of “prey profitability” could be evaluated (e.g., Speed 1993). Having a single measure of profitability would reduce predators’ abilities to juggle their intake of prey containing toxins to avoid the saturation of specific detoxification pathways (Turner and Speed 2001; Halpin et al. 2012), or make more informed decisions based on current state (Kokko et al. 2003; Sherratt 2003; Sherratt et al. 2004; Barnett et al. 2007; Skelhorn and Rowe 2007). However, it could be advantageous for birds in complex foraging environments, where it may be easier to remember less detail about specific prey types and make foraging decisions based on profitability, rather than try to remember specific nutritional and toxicity values for many different species. Knowing how birds, and indeed other predators, learn about and integrate positive and negative outcomes of ingesting toxic prey is important for understanding how foraging decisions on aposematic prey are made, and the effects of those decisions on the evolution of prey defenses.

One final mechanistic explanation for our results is that birds simply learned to discriminate between prey based on their size rather than their visual signals, perhaps because it was a more salient visual cue. This could explain some patterns in our data, such as the fact that birds appeared to preferentially ingest large prey over small prey, regardless of whether they were defended or not (Figure 2). However, birds attacked more small undefended than small defended prey, which could only be on the basis of their visual signals and not their size. Therefore, size discrimination can only be part of the explanation. It is possible that birds initially discriminated on the basis of size, and then in later sessions, learned to discriminate between small undefended and defended prey using the difference in their visual signals. Discrimination between undefended and defended prey types is likely only to occur when it is advantageous for the predator (Skelhorn and Rowe 2006d; Halpin et al. 2012), which could explain why birds only discriminated between the undefended and defended prey when they were small and their nutritional value was relatively low.

Currently, we are unable to discriminate among these mechanistic explanations for our data, and understanding the physiological and cognitive processes that inform foraging decisions on toxic prey will remain a rich seam for future research. However, despite not knowing the exact cognitive mechanism underpinning our results, our findings do have implications for the evolution of prey defenses, and in particular, raise interesting questions about the selection pressures acting on prey signals, toxicity, and body size. Our findings suggest that evolving toxicity may be more beneficial for smaller compared with larger species, and that smaller prey will benefit more from a dose of toxin compared with large bodied prey when the signal size is the same, supporting comparative studies where large prey species appear to be more toxic (Kraemer et al. 2015). What will be interesting in the future is to know the shape of this relationship, and what factors determine investment in defenses according to body size. For example, we don’t know if increasing toxicity may need to scale with body size, and whether prey with the same nutrient:toxin ratio (i.e., toxin concentration) but different body sizes will be predated at equivalent rates. One interesting aspect that has thus far received little attention, is how prey might invest in growth and toxicity. Optimal body size in aposematic prey has mainly been considered in terms of the relative costs and benefits associated with having a larger signal (Higginson and Ruxton 2010): larger signals may be learned more easily by predators (Lindstedt et al. 2008), but also may enhance detectability (Mänd et al. 2007; Remmel and Tammaru 2009). However, toxicity will also be important: our data show that larger bodies require more toxin, which for some species at least, may have a negative impact on body size (e.g., Cohen 1985; Holloway et al. 1993; Zalucki et al. 2001). The interaction between body size, signal size and toxicity is likely to be complex, but our data show that considering the defense of prey is an important factor when thinking about optimal body size of aposematic prey.

This interaction could also have implications for the evolution of mimicry, particularly for Batesian mimics, which copy the patterns of sympatric aposematic models (Bates 1862). Aposematic species that contain more toxin in relation to their nutrient content are perhaps more likely to attract or support Batesian mimics. A recent study compared the body size and toxin content found across populations of an aposematic salamander, *Notophthalmus viridescens*, and found that populations where individuals had larger bodies also contained more toxin (Kraemer et al. 2015). This species also has a Batesian mimic, *Plethodon cinereus*, at some of the locations that were sampled. How well the mimic copied the pattern of its model was affected by the body size but not the toxin content of the model at each location: the pattern of the Batesian mimic was worse and more variable in populations where the body size of the model was larger (Kraemer et al. 2015). Whilst the authors suggest that the increasing signal size of larger bodied models could lead to increased predator avoidance and relaxed selection on mimics, perhaps it could be that there are costs to looking too similar to a large-bodied defended model. It would be intriguing to know whether the relationship between toxicity and body size might be a better predictor of mimic pattern fidelity, or whether differences in body size between model and mimic might also play a role (Marples 1993). Body size is clearly important for Batesian mimicry (Penney et al. 2012), and how this interacts with that of its model is also an intriguing avenue for future research.

In conclusion, our study has demonstrated that a specific dose of toxin does not protect all prey equally, and that the body size of toxic prey plays an important role in the foraging decisions that predators make. This has implications for our understanding of prey defenses, and raises questions about how and what predators learn about toxic prey. Whilst research in prey defenses have tended to focus on the color patterns and the level of toxicity that prey have, it is becoming increasingly clear that we need to start to consider how nutritional value of prey alters predators’ foraging strategies and decisions, and the impact of that on prey defenses and defensive coloration more generally. Whilst understanding the mechanisms by which predators learn about the costs and benefits of eating aposematic prey may seem challenging, it will lead to a more comprehensive understanding of why and when toxicity and aposematism evolves.

## FUNDING

This work was funded by a BBSRC-NERC project grant (BB/G00188X/1).
